# Accurate diagnosis of spinal muscular atrophy and 22q11.2 deletion syndrome using limited deoxynucleotide triphosphates and high-resolution melting

**DOI:** 10.1186/s12864-018-4833-4

**Published:** 2018-06-20

**Authors:** Xiaoqing Zhang, Bo Wang, Lichen Zhang, Guoling You, Robert A. Palais, Luming Zhou, Qihua Fu

**Affiliations:** 1 Department of Laboratory Medicine, Shanghai Children’s Medical Center, Shanghai Jiao Tong University School of Medicine, Shanghai, 200127 People’s Republic of China; 2 Department of Mathematics, Utah Valley University, Orem, UT USA; 3 Department of Pathology, University of Utah Medical School, 50 N. Medical Drive, Salt Lake City, UT 84132 USA

**Keywords:** Copy number variation, High-resolution melting, 22q11.2 deletion syndrome, Spinal muscular atrophy

## Abstract

**Background:**

Copy number variation (CNV) has been implicated in the genetics of multiple human diseases. Spinal muscular atrophy (SMA) and 22q11.2 deletion syndrome (22q11.2DS) are two of the most common diseases which are caused by DNA copy number variations. Genetic diagnostics for these conditions would be enhanced by more accurate and efficient methods to detect the relevant CNVs.

**Methods:**

Competitive PCR with limited deoxynucleotide triphosphates (dNTPs) and high-resolution melting (HRM) analysis was used to detect 22q11.2DS, SMA and SMA carrier status. For SMA, we focused on the copy number of *SMN1* gene. For 22q11.2DS, we analyzed CNV for 3 genes (*CLTCL1, KLHL22*, and *PI4KA*) which are located between different region-specific low copy repeats. *CFTR* was used as internal reference gene for all targets. Short PCR products with separated Tms were designed by uMelt software.

**Results:**

One hundred three clinical patient samples were pretested for possible *SMN1* CNV, including carrier status, using multiplex ligation-dependent probe amplification (MLPA) commercial kit as gold standard. Ninety-nine samples consisting of 56 wild-type and 43 22q11.2DS samples were analyzed for *CLTCL1, KLHL22*, and *PI4KA* CNV also using MLPA. These samples were blinded and re-analyzed for the same CNVs using the limited dNTPs PCR with HRM analysis and the results were completely consistent with MLPA.

**Conclusions:**

Limited dNTPs PCR with HRM analysis is an accurate method for detecting *SMN1* and 22q11.2 CNVs. This method can be used quickly, reliably, and economically in large population screening for these diseases.

**Electronic supplementary material:**

The online version of this article (10.1186/s12864-018-4833-4) contains supplementary material, which is available to authorized users.

## Background

Copy number variation (CNV) refers to excess or deficient copies of portions of a genome, from entire chromosomes (e.g., trisomy) to smaller segments. CNVs are pervasive in the human genome and have been reported to be associated with many genetic disorders [1]. Methods for CNV detection are crucial for identifying genetic risk factors for disease, and carrier status. Several techniques such as comparative genomic hybridization (CGH) microarrays, MLPA, and fluorescent in situ hybridization (FISH) are currently utilized to detect copy number alterations [2, 3]. CGH microarrays are efficient to scan for copy number variation on a large (e.g., genome-wide) scale. FISH is routinely used in the clinical laboratory as a gold standard for detection of CNV. MLPA has been widely used to detect specific CNVs associated with genetic disease in the clinical laboratory [2, 3]. These methods are time-consuming and rely on expensive and specialized instrumentation for operation and analysis.

Competitive PCR with restricted dNTPs and high resolution melting (HRM) is a simple, fast, and economical approach with high accuracy that can be used for targeted evaluation of relative copy number [4]. The necessary multiplex PCR assays can be easily designed using uMELT melting curve prediction software. Spinal muscular atrophy (SMA) and 22q11.2 deletion syndrome (22q11.2DS) are two of the most common genetic diseases which are caused by genomic copy number variations [5, 6]. For affected patients, a definite diagnosis largely relies on genetic diagnosis by the copy number assessment of the *SMN1* gene and the 22q11.2 region, respectively. In this paper we demonstrate the effectiveness of limited dNTPs PCR with HRM analysis for SMA and 22q11.2DS detection.

## Methods

### DNA samples

One hundred three clinical DNA samples from Shanghai Children’s Medical Center exhibiting SMA-related symptoms were analyzed for copy number of *SMN1*. Another 99 clinical DNA samples obtained from Shanghai Children’s Medical Center, 43 having 22q11.2DS and the remaining 56 not having 22q11.2DS were tested for copy number of the 22q11.2 region*.* All identifying data and copy number related data were removed to perform the comparison study of CNV analysis methods in a blinded fashion. Written consent was obtained from parents or guardians of all patients. This study was approved by the Ethics Committee of Shanghai Children’s Medical Center.

### DNA extraction

Peripheral blood samples of all cases were obtained and genomic DNA was extracted by using QIAmp DNA Blood Mini Kit (QIAGEN GmbH, Germany) with standard protocols. DNA was quality tested by optical density 260/280 nm ratios, quantified by UV spectrophotometry, and stored at − 20 °C until use. The DNA concentration of the samples ranged from 30 ng/μl to 214 ng/μl.

### Primer design

Primers for target and reference genes were designed with Primer3 online software (http://bioinfo.ut.ee/primer3-0.4.0/primer3/). Primer specificity was checked on the UCSC in silico PCR (http://genome.ucsc.edu/cgi-bin/hgPcr). The PCR fragment should be unique in human genome, and have at most only rare SNPs or other sequence variants. Human genome databases NCBI (http://www.ncbi.nlm.nih.gov) and UCSC (http://genome.ucsc.edu) were used to check the uniqueness and SNP frequency of PCR sequences. uMelt software (https://dna.utah.edu/umelt/umelt.html) was used to predict the melting temperatures (Tms) of reference and target PCR products, and their melting domain. The reference and target products were well distinguished by melting temperatures. PCR products having only one melting domain were selected. The ΔTm between selected reference and one or more targets were in the necessary range of between 2 °C to 10 °C [4].

### Selection of target genes

SMA is most commonly caused by the homozygous deletion of *SMN1* (0 copies of SMN1). *SMN1* and *SMN2* are highly homologous, having only 5 different nucleotides, that occur on intron 6, exon 7 and on noncoding exon 8. The only coding nucleotide difference is *SMN1* c.840C > T in exon 7 [7]. In the duplex PCR to determine the copy number of *SMN1*, an allele specific primer was used to amplify only *SMN1* c.840C, but not the *SMN2* homolog [4]. Another primer pair amplified a reference segment of *CFTR* exon 7. For SMA, each assay contained 3 control samples, having 2, 1, and 0 copies of *SMN1*, respectively. They were obtained from an SMA non-carrier, an SMA carrier and an affected patient with SMA which had been confirmed by MLPA in our previously study.

**Fig. 1:**
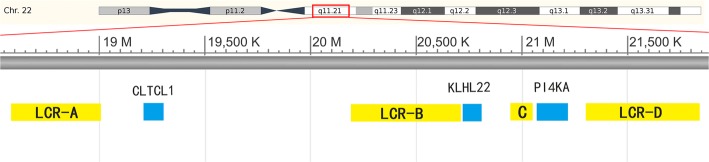
The distribution of *CLTCL1, KLHL22*, and *PI4KA* genes in the 22q11.2 region. *CLTCL1* is located between *LCR22-A* and *LCR22-B. KLHL22* is located between *LCR22-B* and *LCR22-C*. The *PI4KA* gene is located between *LCR22-C* and *LCR22-D*

22q11.2DS is caused by a hemizygous deletion in 22q11.2 region. The deletions start from the LCR22-A region and about 87% extend to LCR22-D, though in some cases they extend only as far as LCR22-B, or LCR22-C [8, 9]. Three genes, from between each pair of adjacent regions, *CLTCL1* from between LCR22-A and LCR22-B, *KLHL22* from between LCR22-B and LCR22-C, and *PI4KA* from between LCR22-C and LCR22-D (Fig. 1) were chosen as targets to detect copy number and determine deletion extent of 22q11.2. For each 22q11.2DS CNV analysis, one duplex PCR and one triplex PCR were performed, both using *CFTR* as copy number reference. The duplex PCR quantified *CLTCL1* copy number, and the triplex PCR simultaneously quantified the copy numbers of *KLHL22* and *PI4KA* genes. The reference and targets primers’ chromosome location, gene name, sequences, Tms and sizes of PCR amplicons are listed in Table 1. All the primers were synthetized by the Beijing Genomics Institute (Beijing, China). For 22q11.2 CNV detection, each assay contained 2 controls, 1 normal sample and 1 sample from a patient having the most frequent 22q11.2 hemizygous deletion, from LCR22-A to LCR22-D.

**Table 1:** Primer Sequences and Data for Reference and Target Genes

Chr.	Region	Primer-Forward	Primer-Reverse	Amplicon Size (bp)	Amplicon Tm (°C)
7	*CFTR* exon 7	TTGTGATTACCTCAGAAATGATTGA	CATTGCTTCTTCCCAGCAGT	68	77.5
5	*SMN1* exon 7	TTCCTTTATTTTCCTTACAGGGTTT	CCTTCCTTCTTTTTGATTTTGTCTG	50	73.5
22	*CLTCL1*	TCAGCTCCTCCAGCTCATCT	TGCATGGATGGACAAGAGTT	82	85
22	*KLHL22*	CTCTCGTTCCGGTGGTACAT	TGATGGAAGCTGAGGTCCTG	100	90
22	*PI4KA*	GCCTGTGGGAGGACAAAATA	TTCTGGCACACCAGTTCATC	97	83

### PCR and high resolution melting

PCR was performed in 10 μl volume containing 0.125 μM–1 μM each primers, 10 mM Tris (pH = 8.3), 0.5 mg/ml BSA, 2 mM MgCl_2_, 0.4 U KlenTaq (Ab Peptides), 6.25 μM dNTPs, 1X LCGreen® Plus dye (BioFire Defense) and 30 ng to 214 ng genomic DNA. Primers concentration varied between reactions to optimize analytical resolution. In duplex PCR, the concentrations of each primer (forward and reverse) were 0.25 μM *CFTR* and 0.5 μM *SMN1*; 0.125 μM *CFTR* and 0.5 μM *CLTCL1*. In the triplex PCR, the concentrations each pair of primers were 0.125 μM *CFTR*, 1 μM *KLHL22* and 0.5 μM *PI4KA*.

PCR and high resolution melting were performed on the Rotor-Gene Q thermocycler (Qiagen GmbH, Germany). PCR conditions were: Denature at 95 °C for 60s, followed by 35 cycles of 95 °C for 10s and 64 °C for 30s. Following amplification, the samples were melted at a 0.3 °C/s melting rate from 65 °C to 95 °C and high-resolution melting data were acquired. To determine the reproducibility of the new method, the PCR and HRM analyses were performed in three independent runs on all samples.

### Analysis

High-resolution melting data were exported and analyzed by MeltWizard 5.1 software [4]. Analysis steps began with exponential background subtraction to remove the fluorescence background, and optional normalization was performed to eliminate the fluorescence differences between samples and correspond to total helicity. Next, melting curves were temperature shifted so that shared intrinsic features were optimally overlaid, so as to remove minor temperature variation between wells. The negative derivative was calculated by degree 2 Savitzky-Golay differentiation. To properly quantify the copy number differences, the reference peaks of all derivative plots were normalized both vertically (to the geometric mean reference peak amplitude) and horizontally (scaling and shifting to the mean temperatures of each peak). After performing unbiased hierarchical clustering in the uniform metric, different copy numbers of the target were visualized and quantified.

### MLPA

MLPA is a copy number quantification method that may be performed using commercially available kits (MRC-Holland, The Netherlands). The P060-B2 kit was used to determine the copy number of *SMN1*. The P250-B2 kit was used to determine the copy number of 22q11.2. All procedures were performed according to the manufacturer’s protocol. After the MLPA reaction, including denaturation, hybridization, ligation and the PCR amplification performing on C1000™ Thermal Cycler (Bio-Rad, USA), the amplified products were separated by electrophoresis on ABI 3130 genetic analyzer (Applied Biosystems, USA). Electrophoresis data were visualized and analyzed with GeneMaker software (SoftGenetics, LLC, USA).

## Results

To verify the effectiveness of limited dNTPs PCR and HRM, 103 clinical samples were tested for SMA, and 99 clinical samples were tested for 22q11.2DS, using a commercial MLPA kit as a gold standard control. Both methods were used to quantify copy numbers of various genes associated with SMA and 22q11.2DS. SMA will be manifested when there are zero copies (homozygous deletion) of the *SMN1* gene. An individual will be classified to be a SMA carrier when there is one copy (heterozygous deletion) of *SMN1*. 22q11.2 deletion syndrome is diagnosed when there are hemizygous deletions of various regions of 22q11.2 from LCR22-A to LCR22-D. Approximately 87% of 22q11.2 deletions span about 3 Mb from LCR22-A to LCR22-D; approximately 8% of deletion span about 1.5 Mb from LCR22-A to LCR22-B; the remaining deletions, approximately 5%, span about 2 Mb from LCR22-A to LCR22-C or about 1.5 Mb LCR22-B to LCR22-D [9]. The 3 genes analyzed were chosen to distinguish these possibilities.

Among the 103 potential SMA samples tested for copy number of *SMN1* exon 7, the MLPA method detected 23 samples having 2 copies (normal), 1 sample having 3 copies (no SMA), 43 samples having 1 copy (SMA carrier) and 36 samples having 0 copies (SMA patient). Among the 99 potential 22q11.2 deletion samples, the MLPA method detected 56 samples having 2 copies (normal), 38 cases having deletions from LCR22-A to LCR22-D; 2 samples having deletions from LCR22-A to LCR22-C; and 3 cases having deletions from LCR22-A to LCR22B.

All samples that were pretested using MLPA for SMA and 22q11.2 associated CNV were blinded prior to performing limited dNTPs PCR and HRM. Copy numbers were quantified by target melting peak heights.

**Fig. 2:**
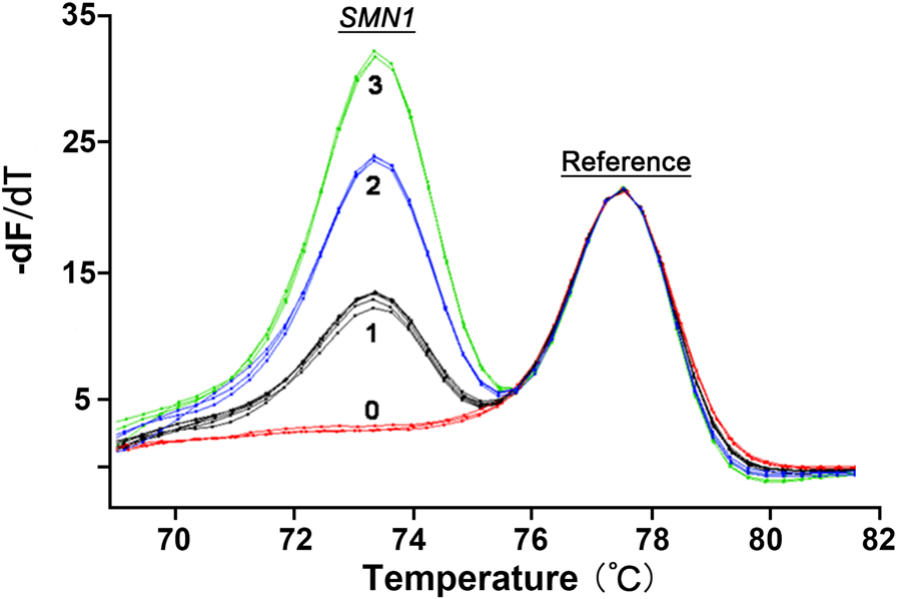
Duplex PCR and HRM result for *SMN1* exon7 copy number assessment. Using the restricted dNTPs and duplex PCR, samples with 3 copies (green), 2 copies (blue), 1 copy (black) and 0 copy (red) of *SMN1* exon7 were well distinguished after normalization against *CFTR* gene. −dF/dT, negative first derivative of fluorescence with respect to temperature

For SMA, the melting peak heights obtained following duplex PCR easily distinguished 2, 1 and 0 copies of *SMN1* (Fig. 2). In 103 blinded potential SMA samples, limited dNTPs PCR and HRM detected 23 samples having 2 copies (normal); 1 sample having more than 2 copies (normal); 43 samples having one copy (carrier, heterozygous deletion) and 36 samples having zero copies (SMA patients, homozygous deletion). Not only were the total number of samples with each copy number the same, but also all individual samples had corresponding copy numbers. For example, the sample exhibiting more than 2 copies by limited dNTPs and HRM was the sample that exhibited 3 copies according to MLPA. There was complete concordance of the results of limited dNTPs PCR and HRM with the results of MLPA (Table 2a, Additional file 1).

**Table 2:** 100% Concordance ^a^ of Limited dNTPs and HRM with MLPA

Target	Copy #	Phenotype	Limited dNTPs and HRM	MLPA
A) *SMN1*	0	SMA	36	36
	1	Carrier	43	43
	2	Normal	23	23
	3	No SMA	1	1
B) 22q11.2	2^b^	Normal	56	56
LCR22A-B	1^c^	22q11.2DS	3	3
LCR22A-C	1^d^	22q11.2DS	2	2
LCR22A-D	1^e^	22q11.2DS	38	38

^a^Individual sample copy numbers were in complete concordance, not just aggregate numbers

^b^2 copies of *CLTCL1, KLHL22* and *PI4KA* genes

^c^1 copy of *CLTCL1* gene. 2 copies of *KLHL22* and *PI4KA* genes

^d^1 copy of *CLTCL1* and *KLHL22* genes. 2 copies of *PI4KA* gene

^e^1 copy of *CLTCL1, KLHL22,* and *PI4KA* genes

**Fig. 3:**
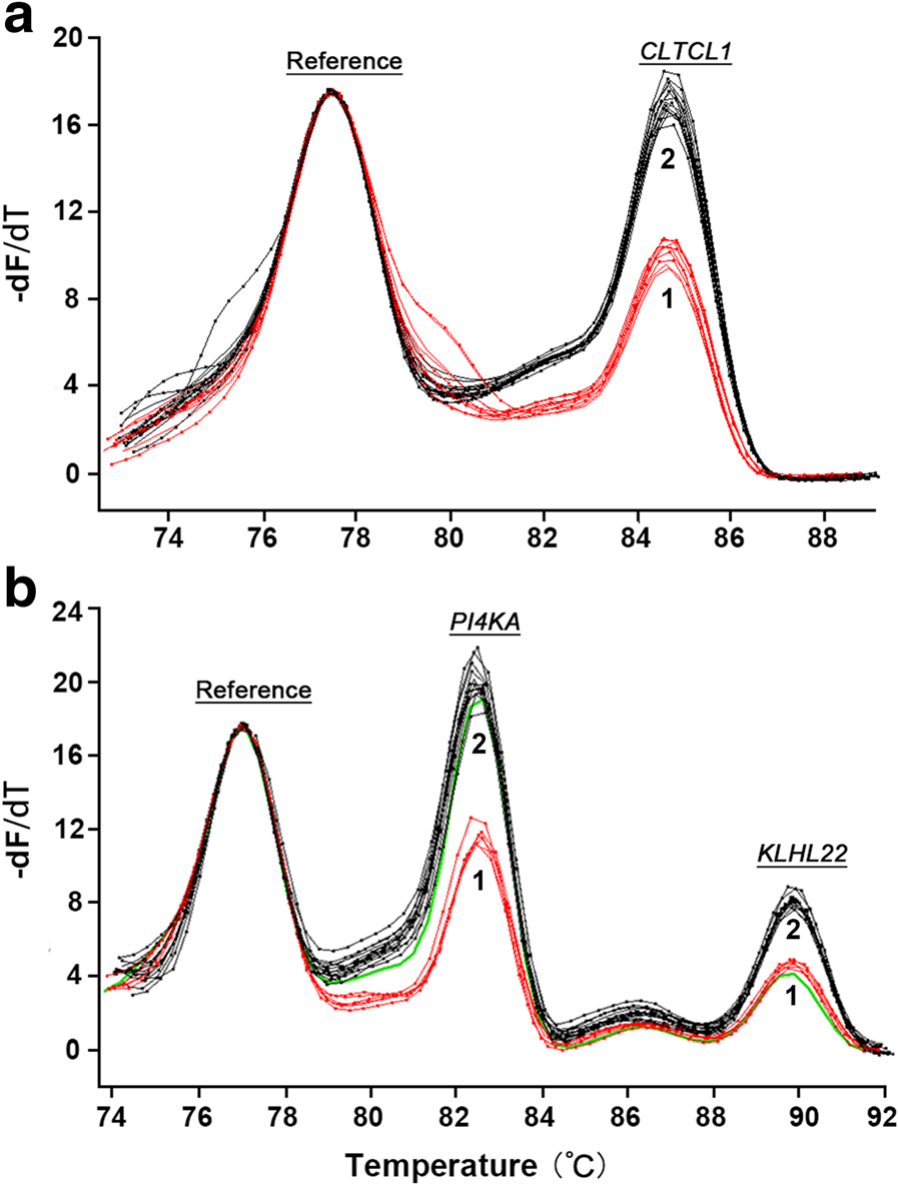
22q11.2 region copy number determination by restricted dNTPs and multiplex PCR. Blinded tests for **a** *CLTCL1*, **b** *PI4KA/KLHL22* are shown. Samples were normalized to the *CFTR* gene. Two copies (Black) and one copy (red) of target genes were well distinguished after normalization against the reference. The green signal in Fig. 3b showed a sample with two copies of *PI4KA* gene and one copy of *KLHL22* gene. −dF/dT, signifies the negative first derivative of fluorescence with respect to temperature

For 22q11.2DS, the melting peak heights obtained following the triplex and duplex PCRs easily distinguished 2 vs. 1 copies of the 3 target genes *CLTCL1, KLHL22,* and *PI4KA* spread across 22q11.2 from LCR22-A to LCR22-D (Fig. 3). In 56 samples 2 copies of the *CLTCL1* gene were found in the duplex reaction (no CNV related 22q11.2DS). In 3 samples, 1 copy of the *CLTCL1* gene was found in the duplex reaction, while 2 copies of the *KLHL22* and *PI4KA* genes were found in the triplex reaction (deletion from LCR22-A to LCR22-B). In 2 samples, 1 copy of the *CLTCL1* gene was found in the duplex reaction, 1 copy of the *KLHL22* gene and 2 copies of the *PI4KA* gene were found in the triplex reaction (deletion from LCR22-A to LCR22-C). In 38 samples, 1 copy of the *CLTCL1* gene was found in the duplex reaction, and 1 copy of the *KLHL22* and *PI4KA* genes were found in the triplex reaction (deletion from LCR22-A to LCR22-D). Not only were the total number of samples with each copy number the same, but also all individual samples had corresponding copy numbers. For example, the 2 samples exhibiting 1 copy of the *KLHL22* gene and 2 copies of the *PI4KA* gene by limited dNTPs and HRM were the same 2 samples having these CNVs according to MLPA. There was complete concordance of the results of limited dNTPs PCR and HRM with the results of MLPA (Table 2b, Additional file 2).

## Discussion

As more CNV associated diseases are being investigated, there is a growing demand for widely applicable, accurate, rapid and economical approaches to discern variations in copy number [10]. Here, we proposed a detection method based on amplification with limited dNTPs and HRM analysis that can be easily adapted to any genomic targets of interest. Careful optimization of primers, conditions, and concentrations are essential so that PCR yields the most informative melting peak data [11]. Besides conventional primer design criteria, additional considerations are required. First, one must aim to design short PCR products in order to prevent the occurrence of multiple melting domains and avoid internal sequence variation. Simultaneously, amplicon sequence differences must result in sufficiently distinct melting temperatures so their peaks can be clearly distinguished in duplex or triplex reactions. The implementation of DNA melting analysis also relies on the instrument resolution. A heating rate of 0.1–1 °C/s is common for high resolution melting devices [12]. For all samples that will be compared in one test, the DNA isolation and purification should be standardized so that they can be dissolved in the same buffer solution. Variable concentrations of salt and different ionic strength will affect the relative peak height of products dramatically.

Above all, the determination of appropriate dNTPs concentration is the most crucial factor for precise relative quantification. When the dNTPs concentration is too low (lower than 1.56 μM), the multiplex PCR may only amplify one target and the shortest amplicon will have the competitive advantage. Above a certain dNTPs concentration (greater than 25 μM), quantification of initial relative reference and target copies are lost in the plateau of primer limitation and no CNV information remains [4]. For each particular CNV assay, an optimal dNTPs concentration can be found at which the amplification is still limited but each copy number is clearly recognizable. Based on these design strategies, we developed assays that illustrate the effectiveness and superior time and cost efficiency of the method in screening for two common CNV diseases, SMA and 22q11.2 deletion syndrome.

SMA is characterized by symmetrical and progressive proximal muscular atrophy owing to the degeneration of α-motor neurons in the anterior horns of spinal cord [13]. Among autosomal recessive disorders, SMA is the most frequent inherited cause of infant and early childhood mortality [14]. Meanwhile, SMA has a high carrier frequency of 1:47 to 1:72 in the general population [15–17]. Most people won’t know their carrier status until they have an affected child [13]. Considering the severity of the disease and the relatively high carrier frequency, timely diagnosis by molecular detection and prevention of new cases by carrier screening are the best ways to help SMA families [18].

*SMN1* produces a full-length SMN protein and is the SMA-determining gene [19]. The homozygous absence of the *SMN1* has been observed in the majority of patients. Direct analysis of *SMN1* gene deletion is valuable in both SMA molecular diagnosis and carrier screening [14, 20]. The quantitative analysis and clinical implications of *SMN1* gene dosage are somewhat complicated by the presence of a highly homologous gene *SMN2* that only differs in five base pairs. Among these differences, a single nucleotide variation of c.840C > T substitution in the coding sequence of exon 7 is pivotal [21]. This site can affect RNA splicing in *SMN2* and produce an unstable and nonfunctional spliced isoform. It is commonly used to differentiate *SMN1* from *SMN2*. In our research, by using allele-specific duplex PCR with limited dNTPs, we can distinguish between the *SMN1* and *SMN2* genes and evaluate the copy number of *SMN1* exon7. Deficient *SMN2* copy number does not cause SMA, so the copy number analysis of *SMN2* it is not routinely performed within the setting of diagnostic or carrier testing for SMA [22]. However, additional *SMN2* copies, that can in rare cases arise in conjunction with missing *SMN1* copies, can partially mitigate the most severe effects of SMA [14, 23, 24]. Followup testing of *SMN2* copy number could be beneficial when screening finds zero copies of *SMN1*.

Our technique can identify the majority SMA patients with homozygous absence of *SMN1* gene and provide universal carrier detection for population based screening. However, this method has limitations. For SMA carrier screening, the silent carriers having two copies of *SMN1* on one chromosome but no one on the other, as well as *SMN1* intragenic mutations could be missed [25]. In this aspect of clinical diagnosis, approximately 5% of affected SMA patients with intragenic mutations in the *SMN1* gene will not be detected by deletion testing methods, including our method and MLPA [26]. Limited dNTPs amplification with HRM is a dosage analysis of the *SMN1* gene copies, while sequence analysis of the *SMN1* gene can be especially useful for the patient who possesses a single copy of *SMN1* yet exhibits an SMA-like phenotype.

The 22q11.2 deletion is the most frequent interstitial deletion in humans with an incidence of 1 in 4000 live births [27]. This region is known to have eight low copy number repeats which make it particularly susceptible to suffer unequal meiotic exchange. The majority of patients have a typical deletion region spanning LCR22-A to LCR22-D, while a smaller portion of patients have a smaller proximal nested deletion extending from LCR22-A to LCR22-B or LCR22-A to LCR22-C [28]. For the detection of 22q11.2 deletion syndrome, two competitive PCRs were performed to obtain a rapid determination of the patient genotype. The use of 3 genes, *CLTCL1*, *KLHL22* and *PI4KA*, located within the deleted regions allowed for discrimination between the various extents of deletion from LCR22-A to LCR22-B, LCR22-C, or LCR22-D. Children with 22q11.2DS display a wide variability in clinical phenotype and experience a host of medical difficulties during early life [29, 30]. When the patients have an identified cause, evaluation of the disease-related features and prevention of certain medical sequelae is feasible and recommendable. Based on previous study, the association of congenital heart disease (CHD) with 22q11.2DS has been well established and it is usually the first presenting symptom in such patients [31]. Molecular screening for 22q11.2 in CHD patients is of great importance in diagnosis and prognosis of disease.

The effectiveness of limited dNTPs amplification with HRM copy number analysis for SMA and 22q11.2DS was validated on clinical samples. Gene dosage results for all cases were in 100% concordance with the results of MLPA, which demonstrated it could be a reliable method for copy number assessment for these patients. In addition, the limited dNTPs method exhibits promising advantages on rapidity, low cost and simple implementation. Firstly, the entire process requires only PCR, melting acquisition and analysis, enabling results to be obtained within 1 h. Secondly, only saturating (high-resolution) fluorescent dye, primers and common PCR components are needed, allowing low reagent cost. Meanwhile, as a closed-tube system, our system requires no additional processing or separation steps, saving considerable labor for operators and greatly reducing the risk of laboratory contamination. Finally, the fluorescence signal can be detected by instrumentation which is readily available in most diagnostic platforms, democratizing the molecular genetic analysis in all kinds of platforms. MLPA has been commonly used for clinical CNV analysis and up to 50 loci can be tested simultaneously in the process. However, MLPA requires at least 2 days for DNA denaturation, probe hybridization and ligation, PCR amplification and data analysis. It demands customized oligonucleotide probes and expensive electrophoresis instruments, both of which increase the cost.

## Conclusions

As a relatively common lethal autosomal recessive disorder with high carrier frequency, SMA copy number testing is an ideal candidate for population-based screening. Considering the high incidence of 22q11.2DS and its close association with congenital heart defects, screening for 22q11.2 deletion is also advisable for all infants with CHD. The rapid and cost-effective detection of CNV in *SMN1* and 22q11.2DS related genes which can be performed and interpreted in all levels of diagnostic platforms are invaluable. Our research demonstrates that limited dNTPs amplification with HRM could be an ideal method for prompt detection of SMA and 22q11.2DS and for SMA carrier screening.

## Additional files

Additional file 1: Restricted dNTPs /HRM and MLPA results for *SMN1* exon7 copy number assessment. **Figure S1-S2.** *SMN1* copy number determination by restricted dNTPs and multiplex PCR. **Figure S3.** MLPA results for normal control, SMA carrier and SMA samples. **Table S1.** Comparison of *SMN1* detection results of Limited dNTPs/HRM and MLPA. (PDF 1052 kb)

Additional file 2: 22q11.2 region copy number determination by restricted dNTPs/HRM and MLPA. **Figure S4.** *CLTCL1* copy number determination by restricted dNTPs and multiplex PCR. **Figure S5.** *PI4KA/KLHL22* copy number determination by restricted dNTPs and multiplex PCR. **Figure S6.** MLPA results for normal control and 22q11.2 deletion samples. **Table S2.** Comparison of 22q11.2 detection results of Limited dNTPs/HRM and MLPA. (PDF 1098 kb)

## Abbreviations

22q11.2DS: 22q11.2 deletion syndrome
CGH: Comparative genomic hybridization arrays
CHD: Congenital heart diseases
CNV: Copy number variation
dNTPs: Deoxynucleotide triphosphates
FISH: Fluorescent in situ hybridization
HRM: High-resolution melting
LCR: Low copy repeat
MLPA: Multiplex ligation-dependent probe amplification
SMA: Spinal muscular atrophy
Tm: Melting temperature

## References

[1] Redon R, Ishikawa S, Fitch KR, Feuk L, Perry GH, Andrews TD, Fiegler H, Shapero MH, Carson AR, Chen W (2006). Global variation in copy number in the human genome. Nature.

[2] Carter NP (2007). Methods and strategies for analyzing copy number variation using DNA microarrays. Nat Genet.

[3] Shen Y, Wu B-L (2009). Designing a simple multiplex ligation-dependent probe amplification (MLPA) assay for rapid detection of copy number variants in the genome. J Genet Genomics.

[4] Zhou L, Palais RA, Paxton CN, Geiersbach KB, Wittwer CT (2015). Copy number assessment by competitive PCR with limiting deoxynucleotide triphosphates and high-resolution melting. Clin Chem.

[5] Merikangas AK, Corvin AP, Gallagher L (2009). Copy-number variants in neurodevelopmental disorders: promises and challenges. Trends Genet.

[6] McDonald-McGinn DM, Sullivan KE, Marino B, Philip N, Swillen A, Vorstman JA, Zackai EH, Emanuel BS, Vermeesch JR, Morrow BE (2015). 22q11.2 deletion syndrome. Nat Rev Dis Primers.

[7] Monani UR, Lorson CL, Parsons DW, Prior TW, Androphy EJ, Burghes AH, JD MP (1999). A single nucleotide difference that alters splicing patterns distinguishes the SMA gene SMN1 from the copy gene SMN2. Hum Mol Genet.

[8] Zhang X, Xu Y, Liu D, Geng J, Chen S, Jiang Z, Fu Q, Sun K (2015). A modified multiplex ligation-dependent probe amplification method for the detection of 22q11.2 copy number variations in patients with congenital heart disease. BMC Genomics.

[9] Shaikh TH, Kurahashi H, Saitta SC, O'Hare AM, Hu P, Roe BA, Driscoll DA, McDonald-McGinn DM, Zackai EH, Budarf ML (2000). Chromosome 22-specific low copy repeats and the 22q11.2 deletion syndrome genomic organization and deletion endpoint analysis. Hum Mol Genet.

[10] Marcinkowska-Swojak MUB, Figlerowicz M, Kozlowski P (2013). An MLPA-based strategy for discrete CNV genotyping: CNV-miRNAs as an example. Hum Mutat.

[11] Erali M, Wittwer CT (2010). High resolution melting analysis for gene scanning. Methods.

[12] Zhou L, Wang L, Palais R, Pryor R, Wittwer CT (2005). High-resolution DNA melting analysis for simultaneous mutation scanning and genotyping in solution. Clin Chem.

[13] Smith M, Calabro V, Chong B, Gardiner N, Cowie S, du Sart D (2007). Population screening and cascade testing for carriers of SMA. Eur J Hum Genet.

[14] Prior TW, Nagan N (2016). Spinal Muscular Atrophy: Overview of Molecular Diagnostic Approaches. Curr Protoc Hum Genet.

[15] Hendrickson BC, Donohoe C, Akmaev VR, Sugarman EA, Labrousse P, Boguslavskiy L, Flynn K, Rohlfs EM, Walker A, Allitto B (2009). Differences in SMN1 allele frequencies among ethnic groups within North America. J Med Genet.

[16] Sugarman EA, Nagan N, Zhu H, Akmaev VR, Zhou Z, Rohlfs EM, Flynn K, Hendrickson BC, Scholl T, Sirko-Osadsa DA (2012). Pan-ethnic carrier screening and prenatal diagnosis for spinal muscular atrophy: clinical laboratory analysis of >72,400 specimens. Eur J Hum Genet.

[17] Wirth B (2000). An update of the mutation spectrum of the survival motor neuron gene (SMN1) in the autosomal recessive spinal muscular atrophy (SMA). Hum Mutat.

[18] Ben-Shachar S, Orr-Urtreger A, Bardugo E, Shomrat R, Yaron Y (2011). Large-scale population screening for spinal muscular atrophy: clinical implications. Genet Med.

[19] Lefebvre S, Bürglen L, Reboullet S, Clermont O, Burlet P, Viollet L, Benichou B, Cruaud C, Millasseau P, Zeviani M (1995). Identification and characterization of a spinal muscular atrophy-determining gene. Cell.

[20] Miskovic M, Lalic T, Radivojevic D, Cirkovic S, Ostojic S, Guc-Scekic M (2014). Ten years of experience in molecular prenatal diagnosis and carrier testing for spinal muscular atrophy among families from Serbia. Int J Gynaecol Obstet.

[21] Lorson CLHE, Androphy EJ, Wirth B (1999). A single nucleotide in the SMN gene regulates splicing and is responsible for spinal muscular atrophy. Proc Natl Acad Sci U S A.

[22] Prior TW, Nagan N, Sugarman EA, Batish SD, Braastad C (2011). Technical standards and guidelines for spinal muscular atrophy testing. Genet Med.

[23] Feldkötter MSV, Wirth R, Wienker TF, Wirth B (2002). Quantitative analyses of SMN1 and SMN2 based on real-time lightCycler PCR: fast and highly reliable carrier testing and prediction of severity of spinal muscular atrophy. Am J Hum Genet.

[24] Calucho M, Bernal S, Alias L, March F, Vencesla A, Rodriguez-Alvarez FJ, Aller E, Fernandez RM, Borrego S, Millan JM (2018). Correlation between SMA type and SMN2 copy number revisited: an analysis of 625 unrelated Spanish patients and a compilation of 2834 reported cases. Neuromuscul Disord.

[25] Ar Rochmah M, Awano H, Awaya T, Harahap NIF, Morisada N, Bouike Y, Saito T, Kubo Y, Saito K, Lai PS (2017). Spinal muscular atrophy carriers with two SMN1 copies. Brain and Development.

[26] Feng Y, Ge X, Meng L, Scull J, Li J, Tian X, Zhang T, Jin W, Cheng H, Wang X (2017). The next generation of population-based spinal muscular atrophy carrier screening: comprehensive pan-ethnic SMN1 copy-number and sequence variant analysis by massively parallel sequencing. Genet Med.

[27] Oskarsdóttir S, Vujic M, Fasth A (2004). Incidence and prevalence of the 22q11 deletion syndrome: a population-based study in Western Sweden. Arch Dis Child.

[28] Burnside RD (2015). 22q11.21 deletion syndromes: a review of proximal, central, and distal deletions and their associated features. Cytogenet Genome Res.

[29] Fung WL, Butcher NJ, Costain G, Andrade DM, Boot E, Chow EW, Chung B, Cytrynbaum C, Faghfoury H, Fishman L (2015). Practical guidelines for managing adults with 22q11.2 deletion syndrome. Genet Med.

[30] Pretto D, Maar D, Yrigollen CM, Regan J, Tassone F (2015). Screening newborn blood spots for 22q11.2 deletion syndrome using multiplex droplet digital PCR. Clin Chem.

[31] McDonald-McGinn DM, Zackai EH (2008). Genetic counseling for the 22q11.2 deletion. Dev Disabil Res Rev.

