# Supplementary material for: Accurate diagnosis of spinal muscular atrophy and 22q11.2 deletion syndrome using limited deoxynucleotide triphosphates and high-resolution melting
Source: BMC Genomics. 2018 Jun 20;19:485. doi: 10.1186/s12864-018-4833-4 (PMC6011344; doi:10.1186/s12864-018-4833-4)
Supplement: Supplementary file 1 — Restricted dNTPs /HRM and MLPA results for SMN1 exon7 copy number assessment. Figure S1-S2. SMN1 copy number determination by restricted dNTPs and multiplex PCR. Figure S3. MLPA results for normal control, SMA carrier and SMA samples. Table S1. Comparison of SMN1 detection results of Limited dNTPs/HRM and MLPA. (PDF 1052 kb) [file 12864_2018_4833_MOESM1_ESM.pdf]

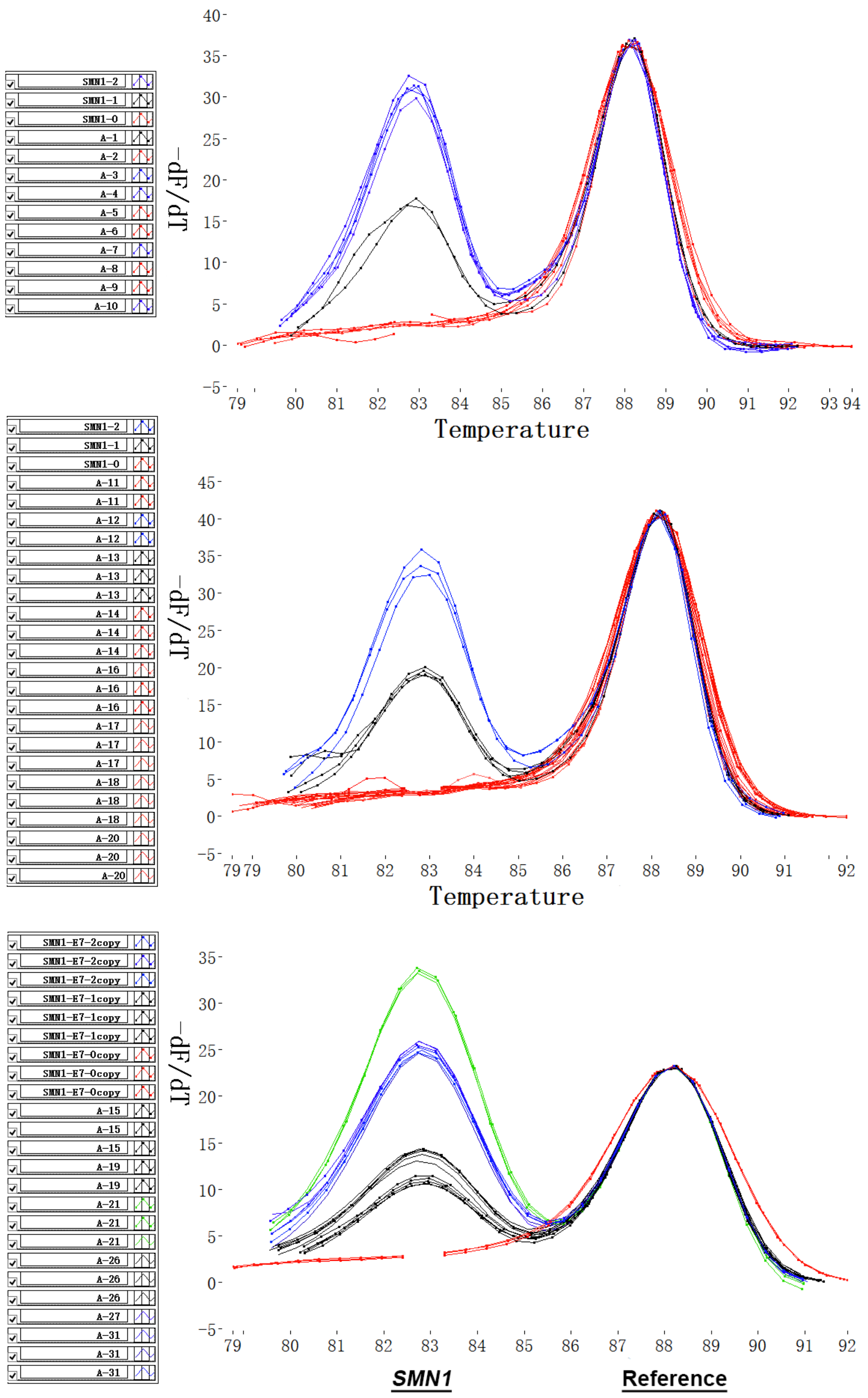

Figure S1. *SMN1* copy number determination by restricted dNTPs and multiplex PCR.
Three copies (green), two copies (blue), one copy (black) and zero copy (red) of target gene were distinguished after normalization against the reference.

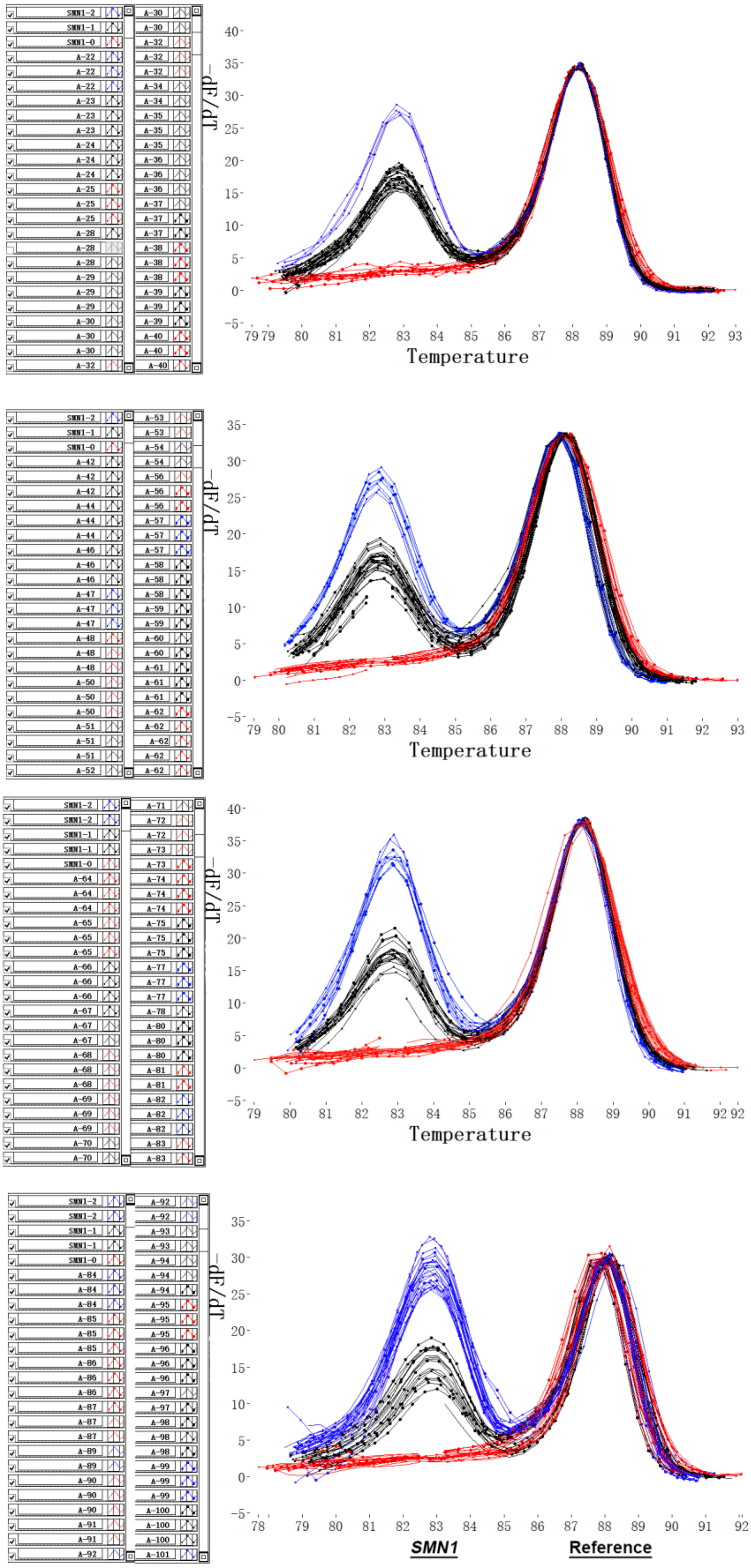

Figure S2. *SMN1* copy number determination by restricted dNTPs and multiplex PCR.

Two copies (blue), one copy (black) and zero copy (red) of target gene were distinguished after normalization against the reference.

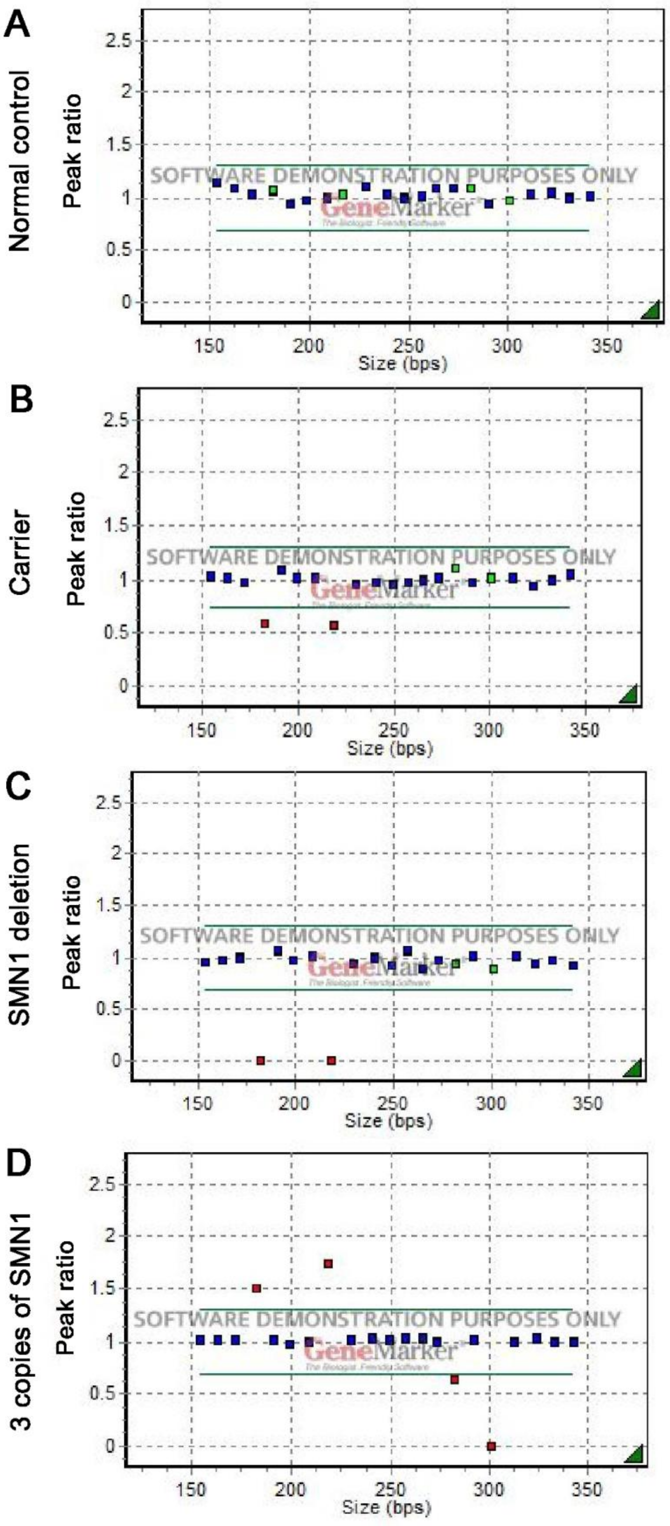

1	**Ctrl_01q32**	208.9	1.000
2	**Ctrl_03p14**	162.0	1.084
3	**Ctrl_03q25**	290.3	0.949
4	**Ctrl_04q25**	171.2	1.027
5	**Ctrl_04q35**	271.9	1.077
6	**Ctrl_05q35**	153.7	1.132
7	**Ctrl_06p22**	310.7	1.028
8	**Ctrl_06q12**	341.0	1.006
9	**Ctrl_08q13**	330.8	0.998
10	**Ctrl_08q24**	322.0	1.051
11	**Ctrl_09q34**	256.2	1.015
12	**Ctrl_10q26**	263.7	1.085
13	**Ctrl_11p13**	198.9	0.976
14	**Ctrl_11q22**	191.0	0.946
15	**Ctrl_12q23**	238.2	1.024
16	**Ctrl_15q13**	247.2	0.996
17	**Ctrl_20p12**	228.7	1.102
18	SMN1_ex07	182.0	1.067
19	SMN1_ex08	217.3	1.038
20	SMN2_ex07	281.1	1.080
21	SMN2_ex08	300.8	0.983

1	**Ctrl_01q32**	209.4	1.012
2	**Ctrl_03p14**	162.9	1.009
3	**Ctrl_03q25**	291.2	0.982
4	**Ctrl_04q25**	171.8	0.978
5	**Ctrl_04q35**	273.1	1.016
6	**Ctrl_05q35**	154.3	1.031
7	**Ctrl_06p22**	311.9	1.006
8	**Ctrl_06q12**	342.1	1.051
9	**Ctrl_08q13**	332.2	1.000
10	**Ctrl_08q24**	323.0	0.943
11	**Ctrl_09q34**	257.4	0.980
12	**Ctrl_10q26**	265.3	0.995
13	**Ctrl_11p13**	199.6	1.005
14	**Ctrl_11q22**	191.7	1.081
15	**Ctrl_12q23**	239.9	0.980
16	**Ctrl_15q13**	249.1	0.956
17	**Ctrl_20p12**	230.0	0.961
18	SMN1_ex07	182.7	0.587
19	SMN1_ex08	218.6	0.572
20	SMN2_ex07	282.1	1.091
21	SMN2_ex08	301.0	1.010

1	**Ctrl_01q32**	209.3	1.006
2	**Ctrl_03p14**	162.8	0.975
3	**Ctrl_03q25**	291.0	1.012
4	**Ctrl_04q25**	171.7	1.001
5	**Ctrl_04q35**	273.0	0.976
6	**Ctrl_05q35**	154.2	0.969
7	**Ctrl_06p22**	312.7	1.020
8	**Ctrl_06q12**	341.9	0.937
9	**Ctrl_08q13**	331.9	0.976
10	**Ctrl_08q24**	322.7	0.946
11	**Ctrl_09q34**	257.3	1.069
12	**Ctrl_10q26**	265.1	0.900
13	**Ctrl_11p13**	199.4	0.983
14	**Ctrl_11q22**	191.6	1.072
15	**Ctrl_12q23**	239.7	1.005
16	**Ctrl_15q13**	248.9	0.936
17	**Ctrl_20p12**	229.9	0.953
18	SMN1_ex07	182.6	0.000
19	SMN1_ex08	218.4	0.000
20	SMN2_ex07	282.0	0.953
21	SMN2_ex08	300.9	0.890

1	**Ctrl_01q32**	209.4	0.991
2	**Ctrl_03p14**	162.9	1.010
3	**Ctrl_03q25**	291.6	1.009
4	**Ctrl_04q25**	171.8	1.022
5	**Ctrl_04q35**	273.6	0.997
6	**Ctrl_05q35**	154.4	1.021
7	**Ctrl_06p22**	312.6	0.989
8	**Ctrl_06q12**	342.3	0.992
9	**Ctrl_08q13**	332.8	0.989
10	**Ctrl_08q24**	323.5	1.029
11	**Ctrl_09q34**	257.6	1.028
12	**Ctrl_10q26**	265.7	1.024
13	**Ctrl_11p13**	199.7	0.983
14	**Ctrl_11q22**	191.9	1.011
15	**Ctrl_12q23**	240.2	1.029
16	**Ctrl_15q13**	249.5	1.007
17	**Ctrl_20p12**	230.3	1.009
18	SMN1_ex07	182.7	1.509
19	SMN1_ex08	219.0	1.738
20	SMN2_ex07	282.4	0.636
21	SMN2_ex08	301.2	0.000

Figure S3. MLPA results for normal control, SMA carrier and SMA samples.

The MLPA spot figures for normal control with 2 copies of *SMN1*(A), carrier with 1 copy of *SMN1* (B), patient with homozygous absence of *SMN1* gene (C) and a sample with 3 copies of *SMN1* gene (D).

Table S1. Comparison of *SMN1* detection results of Limited dNTPs/HRM and MLPA

samples	*SMN1*(copy)		Patients	*SMN1*(copy)	
	HRM	MLPA		HRM	MLPA
A-01	1	1	A-54	1	1
A-02	0	0	A-56	0	0
A-05	0	0	A-58	1	1
A-06	0	0	A-59	1	1
A-08	0	0	A-60	1	1
A-09	0	0	A-61	1	1
A-11	0	0	A-62	0	0
A-13	1	1	A-63	1	1
A-14	0	0	A-64	0	0
A-15	1	1	A-65	0	0
A-16	0	0	A-66	1	1
A-17	0	0	A-67	1	1
A-18	0	0	A-68	0	0
A-19	1	1	A-69	0	0
A-20	0	0	A-70	1	1
A-23	1	1	A-71	1	1
A-24	1	1	A-72	0	0
A-25	0	0	A-73	0	0
A-26	1	1	A-74	0	0
A-28	1	1	A-75	1	1
A-29	1	1	A-76	1	1
A-30	1	1	A-78	1	1
A-32	0	0	A-80	1	1
A-34	1	1	A-81	0	0
A-35	1	1	A-83	0	0
A-36	1	1	A-85	0	0
A-37	1	1	A-86	0	0
A-38	0	0	A-87	0	0
A-39	1	1	A-88	1	1
A-40	0	0	A-90	0	0
A-42	1	1	A-91	0	0
A-43	1	1	A-93	1	1
A-44	1	1	A-94	1	1
A-46	1	1	A-95	0	0
A-48	0	0	A-96	1	1
A-50	0	0	A-97	1	1
A-51	1	1	A-98	1	1
A-52	1	1	A-100	1	1
A-53	0	0	A-104	1	1
