# Supplementary material for: Accurate diagnosis of spinal muscular atrophy and 22q11.2 deletion syndrome using limited deoxynucleotide triphosphates and high-resolution melting
Source: BMC Genomics. 2018 Jun 20;19:485. doi: 10.1186/s12864-018-4833-4 (PMC6011344; doi:10.1186/s12864-018-4833-4)
Supplement: Supplementary file 2 — 22q11.2 region copy number determination by restricted dNTPs/HRM and MLPA. Figure S4. CLTCL1 copy number determination by restricted dNTPs and multiplex PCR. Figure S5. PI4KA/KLHL22 copy number determination by restricted dNTPs and multiplex PCR. Figure S6. MLPA results for normal control and 22q11.2 deletion samples. Table S2. Comparison of 22q11.2 detection results of Limited dNTPs/HRM and MLPA. (PDF 1098 kb) [file 12864_2018_4833_MOESM2_ESM.pdf]

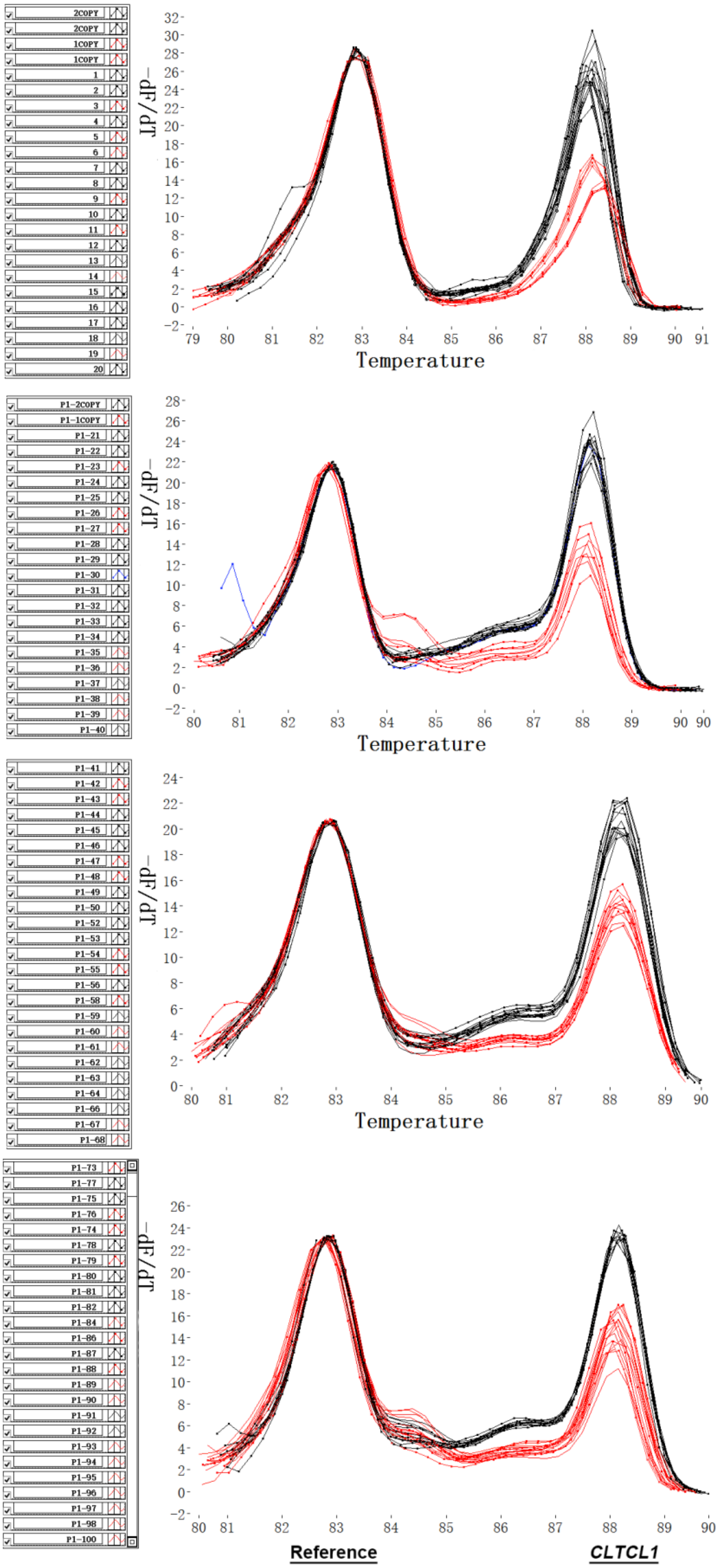

Figure S4. *CLTCL1* copy number determination by restricted dNTPs and multiplex PCR.
Two copies (Black) and one copy (red) of target genes were distinguished after normalization against the reference.

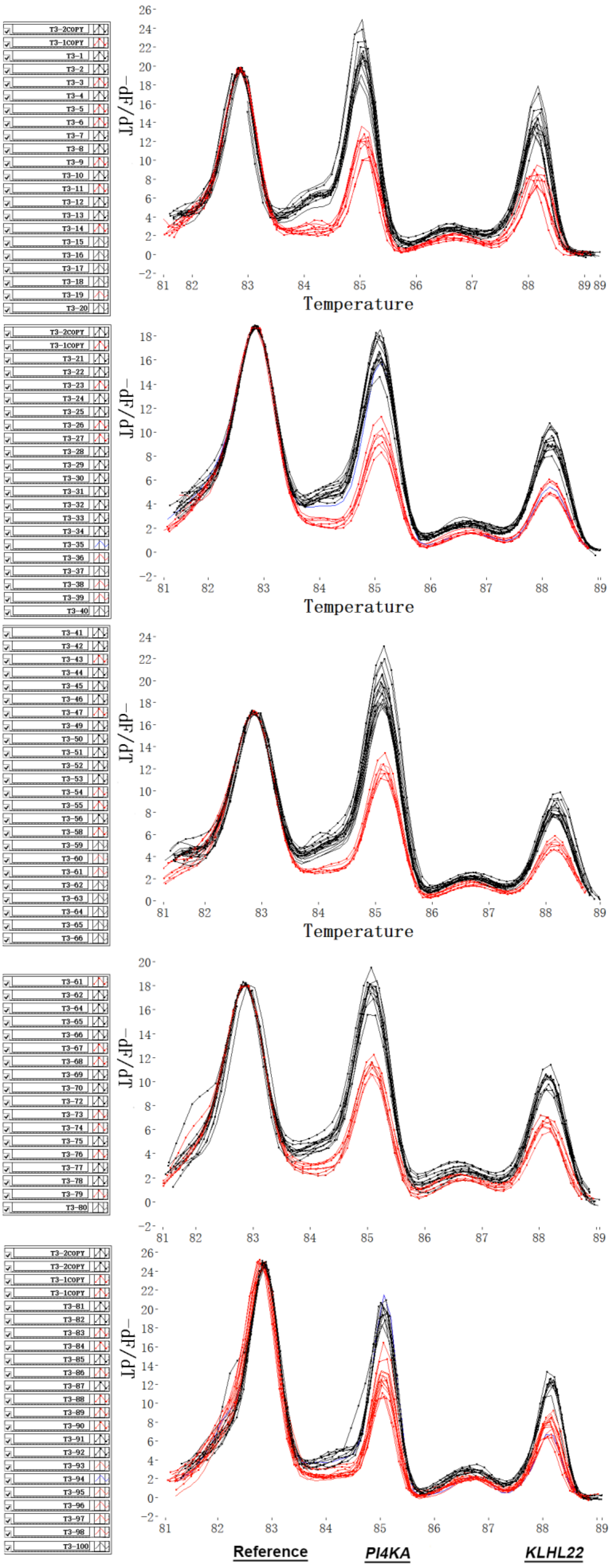

Figure S5. *PI4KA/KLHL22* copy number determination by restricted dNTPs and multiplex PCR.

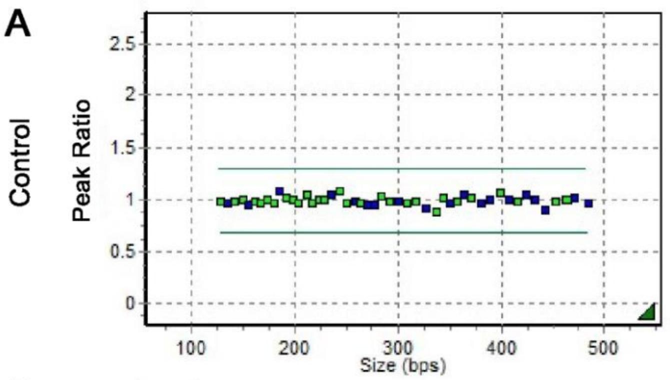

f_CLTCL1	191.3	1.034
g_HIRA	159.9	0.997
h_CDC45L	462.3	1.012
i_CLDN5	196.8	1.021
j_GP1BB	210.1	1.064
k_TBX1_1	172.6	1.012
l_TBX1_2	249.8	0.980
m_TXNRD2	241.9	1.101
n_DGCR8	148.0	1.005
o_ZNF74	202.9	0.981
p_KLHL22	283.4	1.043
q_MED15	316.8	1.000
r_SNAP29	370.8	1.030
s_LZTR1	416.4	1.002

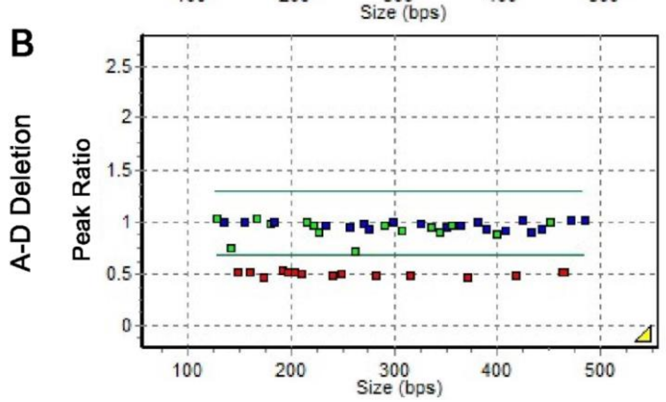

f_CLTCL1	191.2	0.539
g_HIRA	159.4	0.517
h_CDC45L	462.8	0.529
i_CLDN5	196.3	0.528
j_GP1BB	209.8	0.506
k_TBX1_1	172.6	0.476
l_TBX1_2	247.7	0.500
m_TXNRD2	240.2	0.489
n_DGCR8	147.6	0.524
o_ZNF74	202.4	0.527
p_KLHL22	282.1	0.497
q_MED15	315.7	0.488
r_SNAP29	371.3	0.483
s_LZTR1	417.3	0.488

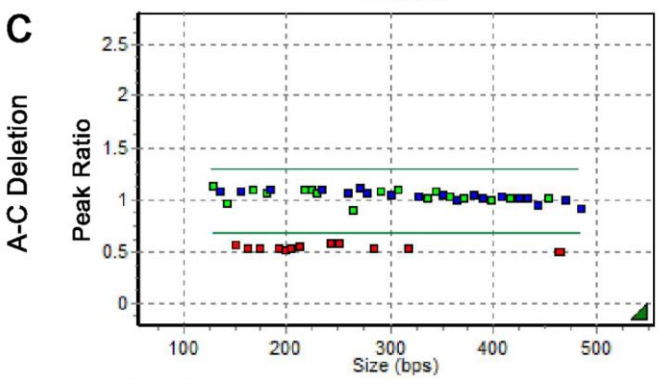

f_CLTCL1	191.4	0.549
g_HIRA	160.0	0.542
h_CDC45L	462.5	0.506
i_CLDN5	196.8	0.533
j_GP1BB	210.2	0.561
k_TBX1_1	172.8	0.549
l_TBX1_2	249.5	0.597
m_TXNRD2	241.6	0.585
n_DGCR8	148.0	0.583
o_ZNF74	202.9	0.543
p_KLHL22	283.0	0.545
q_MED15	316.4	0.544
r_SNAP29	371.0	1.029
s_LZTR1	416.8	1.024

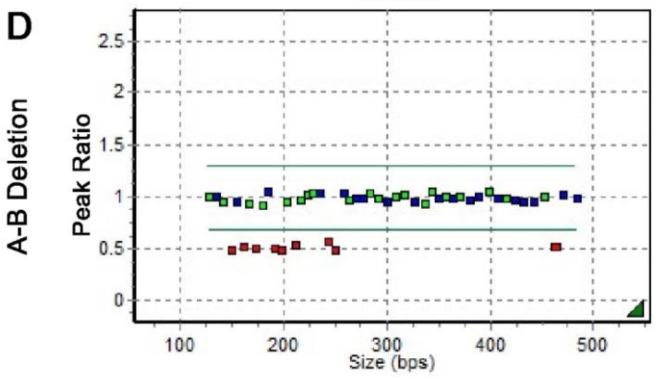

f_CLTCL1	191.3	0.504
g_HIRA	159.9	0.527
h_CDC45L	462.3	0.528
i_CLDN5	196.8	0.488
j_GP1BB	210.1	0.535
k_TBX1_1	172.6	0.505
l_TBX1_2	249.8	0.489
m_TXNRD2	241.9	0.579
n_DGCR8	148.0	0.498
o_ZNF74	202.9	0.955
p_KLHL22	283.4	1.042
q_MED15	316.8	1.026
r_SNAP29	370.8	1.009
s_LZTR1	416.4	0.990

Figure S6. MLPA results for normal control and 22q11.2 deletion samples.

The MLPA spot figures for normal sample (A), patient with LCR22-A to LCR22-D deletion (B), patient with LCR22-A to LCR22-C deletion (C) and patient with LCR22-A to LCR22-B deletion (D).

Table S2. Comparison of 22q11.2 detection results of Limited dNTPs/HRM and MLPA

samples	HRM(copy)			MLPA(copy)		
	CLTCL1	*PI4KA*	*KLHL22*	*CLTCL1*	*PI4KA*	*KLHL22*
3	1	1	1	1	1	1
5	1	1	1	1	1	1
6	1	1	1	1	1	1
9	1	1	1	1	1	1
11	1	1	1	1	1	1
14	1	1	1	1	1	1
19	1	1	1	1	1	1
23	1	1	1	1	1	1
26	1	1	1	1	1	1
27	1	1	1	1	1	1
35	1	2	1	1	2	1
36	1	1	1	1	1	1
38	1	1	1	1	1	1
39	1	1	1	1	1	1
42	1	2	2	1	2	2
43	1	1	1	1	1	1
47	1	1	1	1	1	1
48	1	1	1	1	1	1
54	1	1	1	1	1	1
55	1	1	1	1	1	1
58	1	1	1	1	1	1
60	1	1	1	1	1	1
61	1	1	1	1	1	1
65	1	2	2	1	2	2
67	1	1	1	1	1	1
68	1	1	1	1	1	1
73	1	1	1	1	1	1
74	1	1	1	1	1	1
76	1	1	1	1	1	1
79	1	1	1	1	1	1
83	1	1	1	1	1	1
84	1	1	1	1	1	1
86	1	1	1	1	1	1
88	1	1	1	1	1	1
89	1	1	1	1	1	1
90	1	1	1	1	1	1
93	1	1	1	1	1	1
94	1	2	1	1	2	1
95	1	1	1	1	1	1
96	1	1	1	1	1	1
97	1	1	1	1	1	1
98	1	1	1	1	1	1
100	1	2	2	1	2	2
